# Waterborne polyurethane as a carbon coating for micrometre-sized silicon-based lithium-ion battery anode material

**DOI:** 10.1098/rsos.180311

**Published:** 2018-08-22

**Authors:** Chunfeng Yan, Tao Huang, Xiangzhen Zheng, Cuiran Gong, Maoxiang Wu

**Affiliations:** Key Laboratory of Optoelectronic Materials Chemistry and Physics, Fujian Institute of Research on the Structure of Matter, Chinese Academy of Sciences, Fuzhou 350002, People's Republic of China

**Keywords:** micrometre-sized silicon anodes, water polyurethane, electrochemical performance, 25 and 55°C, lithium-ion battery

## Abstract

Waterborne polyurethane (WPU) is first used as a carbon-coating source for micrometre-sized silicon. The remaining nitrogen (N) and oxygen (O) heteroatoms during pyrolysis of the WPU interact with the surface oxide on the silicon (Si) particles via hydrogen bonding (Si–OH⋯N and Si–OH⋯O). The N and O atoms involved in the carbon network can interact with the lithium ions, which is conducive to lithium-ion insertion. A satisfactory performance of the Si@N, O-doped carbon (Si@CNO) anode is gained at 25 and 55°C. The Si@CNO anode shows stable cycling performance (capacity retention of 70.0% over 100 cycles at 25°C and 60.3% over 90 cycles at 55°C with a current density of 500 mA g^−1^) and a superior rate capacity of 864.1 mA h g^−1^ at 1000 mA g^−1^ (25°C). The improved electrochemical performance of the Si@CNO electrode is attributed to the enhanced electrical conductivity and structural stability.

## 1. Introduction

Lithium-ion batteries (LIBs) are used as an environmentally friendly energy source for consumer electronics and electric vehicles because of their high-energy density [1–5]. Graphite is mainly a negative material for many commercial LIBs and can only provide a maximum theoretical specific capacity of 372 mA h g^−1^; therefore, the capacity of LIBs cannot satisfy high-energy needs [6,7]. Silicon (Si) has received increasing attention as a new negative electrode material with high theoretical capacity (3579 mA h g^−1^) [8]. Also, silicon has a relatively low voltage plateau, and is non-toxic, inexpensive and abundantly available in nature [9–11].

However, Si generally has low electrical conductivity and a large volume change during the lithiation–delithiation process. The volume change can cause bulk Si to be pulverized and lose electrical contact with the current collector, and will also lead to instability of the solid electrolyte interphase (SEI), both of which can lead to fast capacity fading in the LIBs [12–14]. To avoid deterioration of reversible capacity, various nanostructured forms of silicon have been used (e.g. nanoparticles, nanowire and nanoporous structures) [15–17]. These nanostructures can remarkably improve the cycling performance of Si, largely because they can withstand a deep lithiation and delithiation process without cracking. Nevertheless, the nano-Si structure is expensive because of its costly preparation process and low tapping density. Meanwhile, nano-sized Si is easy to reunite because of its large surface area and surface energy, but is difficult to achieve during production in a large-scale application [18]. Compared with nano-sized silicon, micrometre-sized silicon is a good choice [19–21].

In addition to this strategy, surface coating on Si particles and Si–C composites has been attempted to enhance cycling performance. Carbon coatings can reduce volume expansion, increase electrical contact between Si particles and provide a more stable SEI structure [22–24]. Recently, doped carbon with heteroatoms (nitrogen, boron and sulfur) has attracted attention as a promising candidate as a conductive coating agent on electrode materials [22,25,26]. As heteroatoms (N, B) have a similar atomic size, and a higher electronic affinity compared with carbon atoms, the heteroatom-doped carbon layer can form a stronger interaction with the lithium ion. The introduction of a larger heteroatom (e.g. S) can change the structure of the adjacent carbon atoms, improve the large defects of the carbon network and stabilize the amorphous carbon layer structure [26]. Previous reports prove that heteroatom-doped Si–C electrodes have better electrochemical properties with stable electrical conductivity and electrical contact between electrode materials [21,27–29]. These reports are based on studies at room temperature; however, research at an elevated temperature of LIBs is increasingly required, especially in electric vehicle applications. At present, few studies have examined the electrochemical performance of silicon-based anode materials at elevated temperatures [29–31].

In this study, N- and O-doped carbon-coated (Si@CNO) composite is prepared with micrometre-sized silicon and waterborne polyurethane (WPU) as the starting materials using simple physical–chemical methods. The Si@CNO electrode exhibits excellent electrochemical performance at 25 and 55°C. The other N and O heteroatoms in the carbon network during pyrolysis of the WPU with the NHCOO unit interact with the surface oxide on the Si particles through hydrogen bonding (Si–OH⋯N and Si–OH⋯O), which provides a conformal carbon-coated layer and a more stable SEI layer [32]. Moreover, N and O heteroatoms in a carbon layer could interact with lithium ions, which might be beneficial to lithium insertion.

## 2. Material and methods

### 2.1. Materials and experimental procedures

Micrometre-sized silicon powder (approx. 5 μm, Fuzhou Sunout Energy & Material Technology Co. Ltd.) was used as received without further purification. The Si@CNO composites were obtained through a solid–liquid method and a high-temperature cracking method. Micrometre-sized silicon powder (0.1 g) was dispersed in the ethanol solvent (20 ml) under ultrasonication for 30 min and WPU (20 g, approx. 50 wt%) was added to the previous solution. The emulsion was continuously stirred for 3 h. Next, the blend was dried in a blast oven at 100°C for about 2 h to evaporate most of the solvent. The resulting silicon–polyurethane mixture (Si–PU) was kept in a vacuum drying oven at 80°C overnight. Then, the mixture was fired in a tubular furnace at 900°C for 4 h under an argon (Ar) atmosphere. The heating rate was 5°C min^−1^. The composite Si@CNO was taken out after cooling to room temperature under the Ar atmosphere. Finally, the sample was immersed in 5 wt% hydrofluoric acid for 1 h to resolve silicon oxide on the surface of the silicon.

The electrodes were prepared by mixing 80 wt% Si or Si@CNO as the active materials, 10 wt% carbon black and 10 wt% sodium alginate (SA, 2.5 wt%) in deionized water (DI water). The slurry was bladed onto a copper foil, followed by vacuum drying at 90°C overnight. After rolling, the small electrode discs were punched out and weighed. The electrode discs were dried under vacuum at 80°C for 5 h before transferring them to an Ar-filled glove box for cell assembly. Li/Si or Li/Si@CNO cells of 2025 coin type were made in the Ar-filled glove box. The electrolyte used was 1.0 M lithium hexafluorophosphate (LiPF_6_) in ethylene carbonate (EC) and diethyl carbonate (DEC) (3 : 7 wt%) with 10 wt% fluoroethylene carbonate (FEC) as an additive.

### 2.2. Measurements

Instrumental Electrochemical Workstation (VSP, Bio-Logic SAS, France) was employed to execute cyclic voltammetry (CV) on the Si or Si@CNO electrode with a scan rate of 0.1 mV s^−1^ from 1.5 to 0.01 V.

Charge–discharge cycle performance was tested on a Neware battery test system (CT3008). Electrochemical cycling of cells was activated at a charge–discharge of 100 mA g^−1^ and subsequently cycled at a charge–discharge of 500 mA g^−1^, with a potential ranging from 0.01 to 1.5 V.

The cells were also evaluated by electrochemical impedance spectroscopy (EIS) from 100 kHz to 10 mHz under AC stimulus with a 10 mV amplitude (VSP, Bio-Logic SAS, France).

The chemical component of the surface layer on the Si or Si@CNO materials was analysed using X-ray photoelectron spectroscopy (XPS, ESCALAB 250Xi, Thermo Fisher) using an Al Kα line as the X-ray source. The graphite peak at 284.8 eV was used as a reference for the final adjustment of the energy scale in the spectra. Powder X-ray diffraction (XRD) data were collected on a MiniFlex600 diffractometer (Rigaku, Tokyo, Japan), and a scanning electron microscope (SEM, SU8010, Hitachi, Tokyo, Japan) was used to characterize the morphology of the Si and Si@CNO materials. A transmission electron microscope (TEM, JEM-2010, JEOL, Tokyo, Japan) was used to study the details of the micrometre-sized silicon particles coated with N- and O-doped amorphous carbon. The C, H, N and O microanalyses were recorded on an Elementar vario EL III (Langenselbold, Germany) elemental analyser. Thermogravimetric analysis (TGA) was conducted by using a PerkinElmer Diamond TG/DTA instrument (Waltham, MA) at a heating rate of 10°C min^−1^ under air. Raman spectroscopy measurement was conducted with a LabRAM HR Raman spectrometer (Horiba Scientific, Edison, NJ, USA) using a 532 nm excitation wavelength with a 50× microscope objective at a low laser incident power (0.7 mW). Raman shifts were collected in the range of 400–2000 cm^−1^.

## 3. Results and discussion

**Figure 1.**
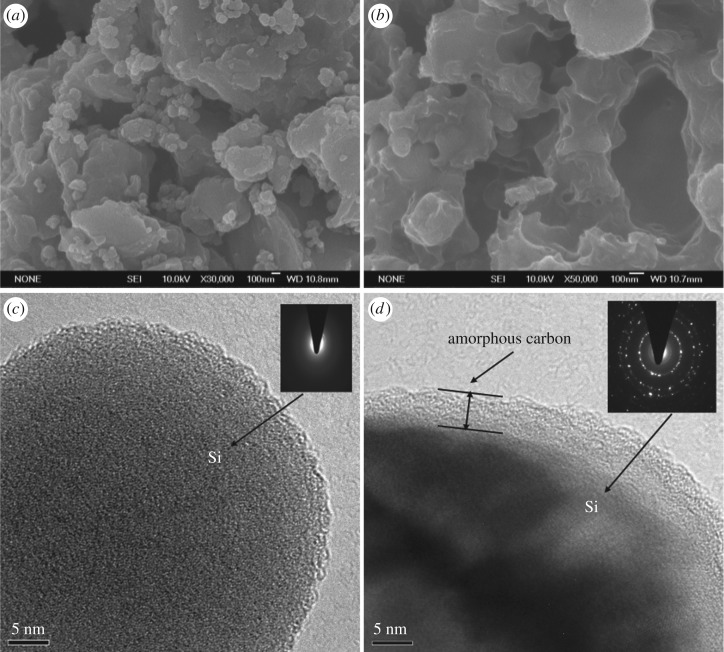
SEM micrographs of (*a*) Si and (*b*) Si@CNO samples. (*c*) TEM image of Si materials with the corresponding SAED pattern, inset of (*c*). (*d*) TEM image exhibiting details of silicon particles coated with amorphous carbon in the Si@CNO samples and the related SAED plot, inset of (*d*).

SEM was used to observe the morphology of commercial micrometre-sized Si and carbon-coated silicon (Si@CNO) composites. The starting material Si surface feature is displayed in the SEM image in figure 1*a*. The particle size is about 3.0–5.0 μm. When compared with figure 1*a*, it is evident that the morphology of composites from the N- and O-doped amorphous carbon coating is quite different from that of pure Si. The carbon-coated composites demonstrate a continuous homogeneous coating layer (figure 1*b*). The detailed morphology of the Si and Si@CNO investigated by TEM is presented in figure 1*c*,*d*. The related selected-area electron-diffraction (SAED) patterns (figure 1*c*,*d*) display indistinct and fine diffraction spots that are characteristic of Si and Si@CNO materials. The SAED patterns demonstrate that the Si samples are amorphous and the Si particles in the Si@CNO materials are crystalline. Meanwhile, the TEM image clearly confirms that the surface of the Si particles is amorphous carbon (figure 1*d*). The coated layer of Si@CNO is approximately 3 nm in thickness.

**Figure 2.**
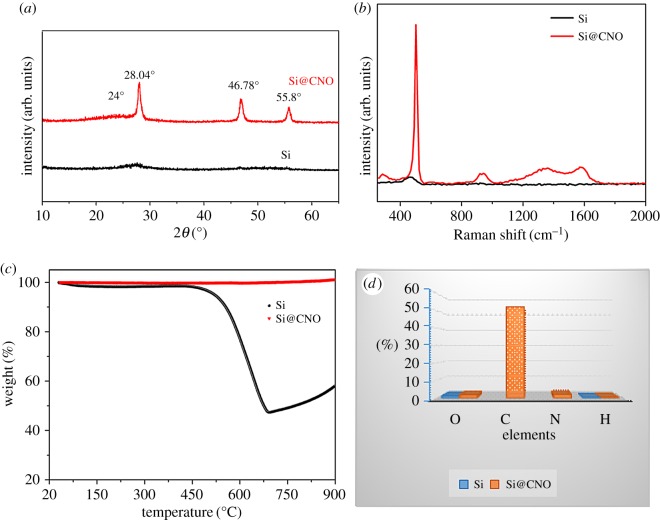
(*a*) XRD patterns, (*b*) Raman spectra, (*c*) TGA curves under an air atmosphere and (*d*) elemental analyser of Si and Si@CNO samples.

The XRD data confirm that the original micrometre-sized silicon particles were amorphous with no significant diffraction peak, whereas the Si@CNO composites were in a crystalline state by cracking at high temperature in the Ar atmosphere (figure 2*a*). The wide carbon peak at about 24° corresponds to amorphous carbon derived from pyrolysis of WPU. The series of peaks at 28.04°, 46.78° and 55.8° demonstrate the cubic crystalline nature of Si. Figure 2*b* presents the Raman spectra of Si and Si@CNO. The two peaks at about 501 and 940 cm^−1^ for the Si@CNO sample indicate the crystalline nature of Si [33]. The two peaks at 1349 cm^−1^ (D band) and 1580 cm^−1^ (G band) are characteristic of the carbon material in Si@CNO [34]. The one wide peak at 470 cm^−1^ of Si indicates that the Si sample is amorphous. This is consistent with the observations of the XRD and SAED patterns. To investigate the percentage of silicon, TGA of the Si and Si@CNO samples is conducted at a rate of 10°C min^−1^ under an air atmosphere. The TGA curve of Si@CNO in figure 2*c* shows that the carbon content is 52.6 wt%. In addition, no obvious weight changes can be observed before 900°C for pure micrometre-sized Si. The elemental analyser in figure 2*d* demonstrates that the carbon content of Si@CNO is 52.0 wt%, which agrees well with the TGA result.

**Figure 3.**
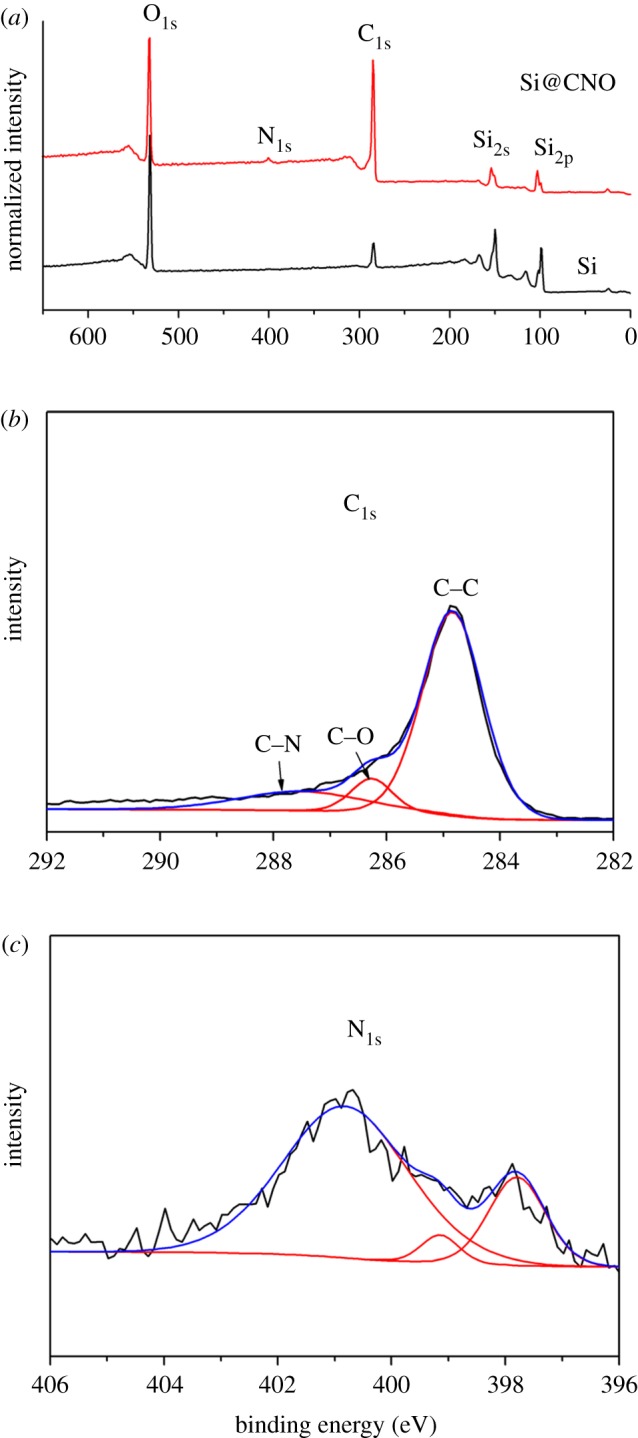
(*a*) XPS patterns of Si and Si@CNO, and XPS spectra of (*b*) C_1s_ and (*c*) N_1s_ for Si@CNO.

The XPS analysis of the Si@CNO sample was executed to define the surface composition of the sample. From the XPS patterns of the Si and Si@CNO samples in figure 3*a*, the peak for 400 eV is the N_1s_ peak, which indicates that the Si@CNO sample contains the nitrogen element. The pure silicon sample, however, does not include this element. The C_1s_ spectra for the Si@CNO sample shown in figure 3*b* indicate three peaks: C–N (287.5 eV), C–O (286.3 eV) and C–C (284.7 eV) [25,35]. Additionally, by employing the elemental analyser (figure 2*d*), the weight ratios of C, N and O in the Si@CNO sample are defined as 52.0, 2.1 and 2.0 wt% (Si sample: O, 1.2 wt%), respectively. From the results of the analysis, the N and O elements are verified to merge into the carbon-coated layer and become covalently bonded with the carbon.

**Figure 4.**
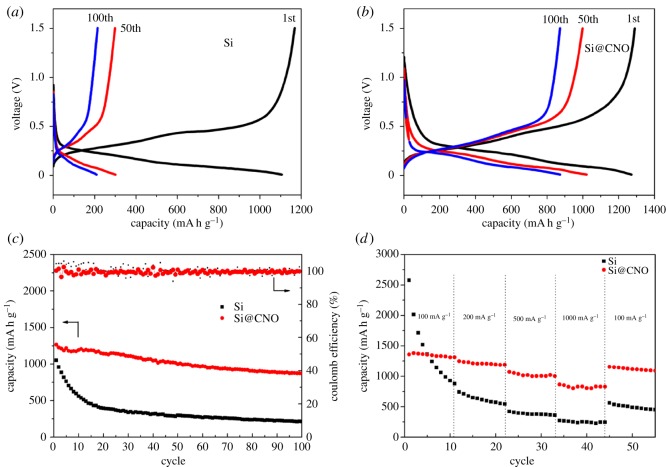
Charge–discharge profiles of the cells with electrodes of (*a*) Si and (*b*) Si@CNO at 1, 50 and 100 cycles at 25°C. (*c*) Capacity retention and coulombic efficiency curves of Si and Si@CNO versus cycle number at 25°C. (*d*) Rate capability profiles of Si and Si@CNO cells cycled at various current densities at 25°C.

To study the electrochemical performance of Si and Si@CNO electrodes, 2025 coin-type cells (Li/Si or Li/Si@CNO) were made to take a series of galvanostatic measurements at 25°C. As expected, the N- and O-doped carbon-coated Si@CNO electrode provides superior cycling and rate performance. For the Li/Si cells, the voltage plateau is shortened and even disappears after 100 cycles (figure 4*a*). This result presumably is due to the large volume expansion of Si and the resulting loss of contact with the current collector, which means that the Si cannot react with Li^+^ and contribute to the capacity after 100 cycles. Instead, the Li/Si@CNO cells reveal the steady voltage profiles in the consecutive cycles (figure 4*b*). The two electrodes both show a voltage plateau at 0.1 V for the Li–Si alloying reaction and 0.45 V for the de-alloying process, which is compatible with the behaviour of Si [35]. This leads to a large irreversible capacity loss and a low initial coulombic efficiency (CE). The Si@CNO electrode exhibits a higher initial CE of 78.5% compared with an initial CE of 77.4% for the Si electrode. This indicates that the N- and O-doped carbon-coated layer can improve the reversible capacity during the first charge–discharge cycle by forming a stable SEI film. Figure 4*c* shows the cycling stability and the CE plots of the Si and Si@CNO electrodes at a charge–discharge current of 500 mA g^−1^ for 100 cycles between 0.01 and 1.5 V. The trend of cycling capacity of the two electrodes, however, is completely different. As displayed in figure 4*c*, after 100 cycles, the capacity retentions of the Li/Si@CNO cells and the Li/Si cells are about 70.0% and 20.4%, respectively. The reversible capacity change of the Li/Si@CNO cells shows a decrease from 1267.2 to 887.1 mA h g^−1^, whereas the capacity of the Li/Si cells drastically fades from 1051.3 to 214.3 mA h g^−1^. The excellent electrochemical performance of the Si@CNO electrode can be attributed to the stable N- and O-doped carbon-coated layer, which increases structural stability and electrical conductivity. The advantage of the N- and O-doped carbon-coated layer is also reflected in the remarkably improved rate capacity of the Si@CNO composite electrode, as shown in figure 4*d*. The rate capabilities of the Si and Si@CNO electrodes are measured at different current densities: 100, 200, 500 and 1000 mA g^−1^. Even at 1000 mA g^−1^, the Li/Si@CNO cells maintain an excellent capacity of 864.1 mA h g^−1^, whereas the capacity of the Li/Si cells rapidly drops to 270 mA h g^−1^. The capacity of the Li/Si@CNO cells is restored quickly to 1155.6 mA g^−1^ when the current comes back to 100 mA g^−1^.

For further LIB application research, the electrochemical performance of Si and Si@CNO electrodes was also considered at elevated temperatures (55°C) (electronic supplementary material, figure S1). At a current density of 500 mA g^−1^, the initial discharge capacity of the Si@CNO electrode is 1550.8 mA h g^−1^, and the discharge capacity is maintained at 665 mA h g^−1^ after 90 cycles. Although the Si electrode shows poor cycling stability, the capacity decreases from 1056 mA h g^−1^ to 148 mA h g^−1^. The capacity retention of the Li/Si@CNO cells and the Li/Si cells is about 60.3% and 13.5%, respectively, after 90 cycles. The obvious difference in cycling stability may depend on the formation of the N- and O-doped carbon layer on the surface of Si to increase structural stability and suppress a large volume change.

**Figure 5.**
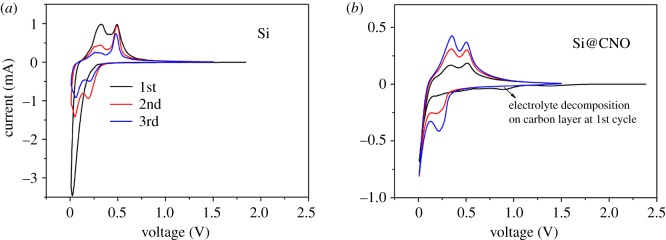
First three cycles of CV plots for the (*a*) Si and (*b*) Si@CNO electrodes between 0.01 and 1.5 V at 0.1 mV s^−1^.

CV measurements were employed to characterize the oxidation–reduction potential of the Si and Si@CNO composite electrodes (figure 5*a*,*b*). It is clearly seen that CV scans of the Si and Si@CNO electrodes are virtually identical and exhibit the characteristic Si anode behaviours. In the first cathodic scan of the Si@CNO electrode, there is a broad cathodic peak at 0.89 V, while it is not found for the Si electrode. The peak has disappeared in the second and third cathodic scans, which could be attributed to electrolyte decomposition on the surface of the carbon-coated layers (the formation process of the SEI film) [25]. Only one sharp peak at approximately 0 V is observed in the first cathodic scan representing the Li-alloying process of crystalline Si to form the amorphous Li*_x_*Si phase [36]. One additional cathodic peak appears at approximately 0.2 V in the second and third cathodic scans, which corresponds to a series of Li–Si alloy formations. In the anodic scan, two peaks located at 0.35 and 0.50 V are related to de-alloying with the Li–Si alloys, and in agreement with the works in the literature regarding silicon-based anode materials.

**Figure 6.**
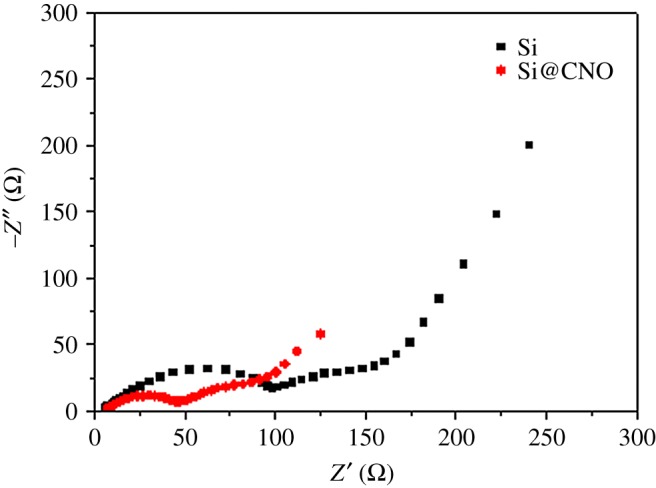
Electrochemical impedance patterns of Si and Si@CNO composite electrodes discharged to 0.01 V after 100 cycles at 25°C.

To further verify the reason for the enhanced performance of the Si and Si@CNO anode, EIS of the electrodes was evaluated after 100 cycles at the full delithiation state at 25°C (figure 6). The semicircle in the high-frequency region reflecting the resistance of the SEI layer (*R*_SEI_) and the semicircle in the middle-frequency region corresponding to the charge-transfer resistance (*R*_ct_) both were observed for the Li/Si and Li/Si@CNO cells [11,37]. After 90 cycles at 55°C, the resistance values of both Si and Si@CNO increased, and the Si@CNO electrode exhibited less increased resistance (electronic supplementary material, figure S2). The numerical gap between the Li/Si and Li/Si@CNO electrodes increased at 55°C. Clearly, the Li/Si cells have a larger *R*_SEI_ and *R*_ct_ than the Li/Si@CNO cells at both 25 and 55°C. Namely, the N- and O-doped carbon-coated layer reduces the resistance values of *R*_SEI_ and *R*_ct_. These results are in keeping with the observation from the previous charge–discharge cycle performance and rate capacity.

## 4. Conclusion

A high-capacity Si@CNO composite material was successfully prepared by cracking a WPU carbon source. An N- and O-doped carbon-coated Si@CNO negative electrode provides superior cycling and rate performance compared to a non-coated micrometre-sized Si electrode. This good electrochemical performance can be credited to the N and O heteroatoms binding to the surface of the Si particles and to the strong interaction between the carbon layer and the Li ion. These interrelations increase the electrical conductivity and structural stability of the Si@CNO negative materials. In addition, the solid–liquid method and the high-temperature cracking process are simple and highly universal in existing battery fabrication technology. These results promote the practical application of Si@CNO composite materials in the manufacture of high-performance Si-based anodes using inexpensive micrometre-sized Si particles.

## Supplementary Material

supplementary material

## Data accessibility

The data supporting this article have been uploaded as part of the electronic supplementary material.

## References

[1] Armand M, Tarascon JM 2008 Building better batteries. Nature 451, 652–657. (10.1038/451652a) 18256660

[2] Li XR et al. 2018 Nitrogen-doped cobalt oxide nanostructures derived from cobalt–alanine complexes for high-performance oxygen evolution reactions. Adv. Funct. Mater. 28, 201800886 (10.1002/adfm.201800886)

[3] Li B, Gu P, Feng YC, Zhang GX, Huang KS, Xue HG, Pang H 2017 Ultrathin nickel-cobalt phosphate 2D nanosheets for electrochemical energy storage under aqueous/solid-state electrolyte. Adv. Funct. Mater. 27, 1605784 (10.1002/adfm.201605784)

[4] Zheng SS, Li XR, Yan BY, Hu Q, Xu YX, Xiao X, Xue HG, Pang H, 2017 Transition-metal (Fe, Co, Ni) based metal-organic frameworks for electrochemical energy storage. Adv. Energy Mater. 7, 201602733 (10.1002/aenm.201602733)

[5] Luo YQ, Tang YJ, Zheng SS, Yan Y, Xue HG, Pang H 2018 Dual anode materials for lithium- and sodium-ion batteries. J. Mater. Chem. A 6, 4236–4259. (10.1039/C8TA00107C)

[6] Evarts EC 2015 Lithium batteries: to the limits of lithium. Nature 526, S93–S95. (10.1038/526S93a) 26509953

[7] Dahn JR, Zheng T, Liu YH, Xue JS 1995 Mechanisms for lithium insertion in carbonaceous materials. Science 270, 590–593. (10.1126/science.270.5236.590)

[8] Etacheri V, Marom R, Elazari R, Salitra G, Aurbach D 2011 Challenges in the development of advanced Li-ion batteries: a review. Energ. Environ. Sci. 4, 3243–3262. (10.1039/C1EE01598B)

[9] Li H, Huang XJ, Chen LQ, Wu ZG, Liang Y 1999 A high capacity nano-Si composite anode material for lithium rechargeable batteries. Electrochem. Solid St. 2, 547–549. (10.1149/1.1390899)

[10] Wu H, Cui Y 2012 Designing nanostructured Si anodes for high energy lithium ion batteries. Nano Today 7, 414–429. (10.1016/j.nantod.2012.08.004)

[11] Yu XH, Yang HY, Meng HW, Sun YL, Zheng J, Ma DQ, Xu XH 2015 Three-dimensional conductive gel network as an effective binder for high-performance Si electrodes in lithium-ion batteries. ACS Appl. Mater. Inter. 7, 15 961–15 967. (10.1021/acsami.5b04058) 26154655

[12] McDowell MT, Ryu I, Lee SW, Wang CM, Nix WD, Cui Y 2012 Studying the kinetics of crystalline silicon nanoparticle lithiation with *in situ* transmission electron microscopy. Adv. Mater. 24, 6034–6041. (10.1002/adma.201202744) 22945804

[13] McDowell MT, Lee SW, Nix WD, Cui Y 2013 25th anniversary article: understanding the lithiation of silicon and other alloying anodes for lithium-ion batteries. Adv. Mater. 25, 4966–4984. (10.1002/adma.201301795) 24038172

[14] Wu H et al. 2012 Stable cycling of double-walled silicon nanotube battery anodes through solid-electrolyte interphase control. Nat. Nanotechnol. 7, 309–314. (10.1038/NNANO.2012.35) 22447161

[15] Zhou M, Li XL, Wang B, Zhang YB, Ning J, Xiao ZC, Zhang XH, Chang YH, Zhi LJ 2015 High-performance silicon battery anodes enabled by engineering graphene assemblies. Nano Lett. 15, 6222–6228. (10.1021/acs.nanolett.5b02697) 26308100

[16] Wang B, Li XL, Qiu TF, Luo B, Ning J, Li J, Zhang XF, Liang MH, Zhi LJ 2013 High volumetric capacity silicon-based lithium battery anodes by nanoscale system engineering. Nano Lett. 13, 5578–5584. (10.1021/nl403231v) 24164145

[17] Kim H, Han B, Choo J, Cho J 2008 Three-dimensional porous silicon particles for use in high-performance lithium secondary batteries. Angew. Chem. Int. Edit. 47, 10 151–10 154. (10.1002/anie.200804355) 19016293

[18] Ren WF, Wang YH, Tan QQ, Zhong ZY, Su FB 2016 Novel silicon/carbon nano-branches synthesized by reacting silicon. with methyl chloride: a high performing anode material in lithium ion battery. J. Power Sources 332, 88–95. (10.1016/j.jpowsour.2016.09.110)

[19] Li C, Shi TF, Yoshitake H, Wang HY 2016 Improved performance in micron-sized silicon anodes by in situ polymerization of acrylic acid-based slurry. J. Mater. Chem. A 4, 16 982–16 991. (10.1039/c6ta05650d)

[20] Sasidharachari K, Na BK, Woo SG, Yoon S, Cho KY 2016 Facile conductive surface modification of Si nanoparticle with nitrogen-doped carbon layers for lithium-ion batteries. J. Solid State Electr. 20, 2873–2878. (10.1007/s10008-016-3291-7)

[21] Roy AK, Zhong MJ, Schwab MG, Binder A, Venkataraman SS, Tomovic Z 2016 Preparation of a binder-free three-dimensional carbon foam/silicon composite as potential material for lithium ion battery anodes. ACS Appl. Mater. Inter. 8, 7343–7348. (10.1021/acsami.5b12026) 26909748

[22] Vrankovic D, Reinold LM, Riedel R, Graczyk-Zajac M 2016 Void-shell silicon/carbon/SiCN nanostructures: toward stable silicon-based electrodes. J. Mater. Sci. 51, 6051–6061. (10.1007/s10853-016-9911-x)

[23] Jiang Y, Chen S, Mu DB, Wu BR, Liu Q, Zhao ZK, Wu F 2017 A three-dimensional network structure Si/C anode for Li-ion batteries. J. Mater. Sci. 52, 10 950–10 958. (10.1007/s10853-017-1253-9)

[24] Jeong MG, Islam M, Du HL, Lee YS, Sun HH, Choi W, Lee JK, Chung KY, Jung HG 2016 Nitrogen-doped carbon coated porous silicon as high performance anode material for lithium-ion batteries. Electrochim. Acta 209, 299–307. (10.1016/j.electacta.2016.05.080)

[25] Shao D, Smolianova I, Tang DP, Zhang LZ 2017 Novel core-shell structured Si/S-doped-carbon composite with buffering voids as high performance anode for Li-ion batteries. RSC Adv. 7, 2407–2414. (10.1039/c6ra26247c)

[26] Zhang YC, You Y, Xin S, Yin YX, Zhang J, Wang P, Zheng XS, Cao FF, Guo YG 2016 Rice husk-derived hierarchical silicon/nitrogen-doped carbon/carbon nanotube spheres as low-cost and high-capacity anodes for lithium-ion batteries. Nano Energy 25, 120–127. (10.1016/j.nanoen.2016.04.043)

[27] Wang X et al. 2014 Atomistic origins of high rate capability and capacity of N-doped graphene for lithium storage. Nano Lett. 14, 1164–1171. (10.1021/nl4038592) 24479759

[28] Etacheri V, Geiger U, Gofer Y, Roberts GA, Stefan IC, Fasching R, Aurbach D 2012 Exceptional electrochemical performance of Si-nanowires in 1,3-dioxolane solutions: a surface chemical investigation. Langmuir 28, 6175–6184. (10.1021/la300306v) 22428945

[29] Park H, Choi S, Lee SJ, Cho YG, Hwang G, Song HK, Choi NS, Park S 2016 Design of an ultra-durable silicon-based battery anode material with exceptional high-temperature cycling stability. Nano Energy 26, 192–199. (10.1016/j.nanoen.2016.05.030)

[30] Park H, Choi S, Lee S, Hwang G, Choi NS, Park S 2015 Novel design of silicon-based lithium-ion battery anode for highly stable cycling at elevated temperature. J. Mater. Chem. A 3, 1325–1332. (10.1039/c4ta05961a)

[31] Wu H, Yu GH, Pan LJ, Liu NA, McDowell MT, Bao ZA, Cui Y 2013 Stable Li-ion battery anodes by *in-situ* polymerization of conducting hydrogel to conformally coat silicon nanoparticles. Nat. Commun. 4, 147 (10.1038/ncomms2941) 23733138

[32] Yan JF, Kraytsberg A, Ein-Eli Y 2015 In-situ Raman spectroscopy mapping of Si based anode material lithiation. J. Power Sources 282, 294–298*.* (10.1016/j.jpowsour.2015.02.044)

[33] Zhang ZL, Wang YH, Ren WF, Tan QQ, Chen YF, Li H, Zhong ZY, Su FB 2014 Scalable synthesis of interconnected porous silicon/carbon composites by the Rochow reaction as high-performance anodes of lithium ion batteries. Angew. Chem. Int. Edit. 53, 5165–5169. (10.1002/anie.201310412) 24700513

[34] Kettle J, Ding Z, Horie M, Smith GC 2016 XPS analysis of the chemical degradation of PTB7 polymers for organic photovoltaics. Org. Electron. 39, 222–228. (10.1016/j.orgel.2016.10.016)

[35] Shao D, Tang DP, Mai YJ, Zhang LZ 2013 Nanostructured silicon/porous carbon spherical composite as a high capacity anode for Li-ion batteries. J. Mater. Chem. A 1, 15 068–15 075. (10.1039/c3ta13616g)

[36] Guo JC, Sun A, Chen XL, Wang CS, Manivannan A 2011 Cyclability study of silicon-carbon composite anodes for lithium-ion batteries using electrochemical impedance spectroscopy. Electrochim. Acta 56, 3981–3987. (10.1016/j.electacta.2011.02.014)

[37] Uchida S, Mihashi M, Yamagata M, Ishikawa M 2015 Electrochemical properties of non-nano-silicon negative electrodes prepared with a polyimide binder. J. Power Sources 273, 118–122. (10.1016/j.jpowsour.2014.09.096)

