# Supplementary material for: Waterborne polyurethane as a carbon coating for micrometre-sized silicon-based lithium-ion battery anode material
Source: R Soc Open Sci. 2018 Aug 22;5(8):180311. doi: 10.1098/rsos.180311 (PMC6124086; doi:10.1098/rsos.180311)
Supplement: supplementary material [file rsos180311supp1.doc]

Supporting information

**Fig. S1** Capacity retention and coulomb efficiency curves of Si and Si@CNO versus cycle number at 55 °C.

**Fig. S2** Impedance spectra of Si and Si@CNO composite electrodes discharged to 0.01 V after 90 cycles at 55 °C.
